# Delineating the role of extracellular vesicles in cancer metastasis: A comprehensive review

**DOI:** 10.3389/fimmu.2022.966661

**Published:** 2022-08-19

**Authors:** Misba Majood, Sonali Rawat, Sujata Mohanty

**Affiliations:** Stem Cell Facility, All India Institute of Medical Sciences, New Delhi, India

**Keywords:** tumor microenvironment (TME), Extracellular vesicles (EVs), biomarker, Carcinogenesis, cargo sorting, liquid biopsy

## Abstract

Extracellular vesicles (EVs) are subcellular messengers that aid in the formation and spread of cancer by enabling tumor-stroma communication. EVs develop from the very porous structure of late endosomes and hold information on both the intrinsic “status” of the cell and the extracellular signals absorbed by the cells from their surroundings. These EVs contain physiologically useful components, including as nucleic acids, lipids, and proteins, which have been found to activate important signaling pathways in tumor and tumor microenvironment (TME) cells, aggravating tumor growth. We highlight critical cell biology mechanisms that link EVS formation to cargo sorting in cancer cells in this review.Sorting out the signals that control EVs creation, cargo, and delivery will aid our understanding of carcinogenesis. Furthermore, we reviewed how cancer development and spreading behaviors are affected by coordinated communication between malignant and non-malignant cells. Herein, we studied the reciprocal exchanges *via* EVs in various cancer types. Further research into the pathophysiological functions of various EVs in tumor growth is likely to lead to the discovery of new biomarkers in liquid biopsy and the development of tumor-specific therapies.

## Introduction

Cancer metastasis is accountable for more than 90% of all cancer-associated fatalities. It is a complicated affair in which cancer spreads from a chief tumor to several organs in the body (1). Metastasis is not a cancer cell-independent action, but somewhat a collaborative endeavor that requires the tumor microenvironment’s assistance. Extensive scientific research has revealed that cancer cells affect several non-malignant cells and extracellular matrix (ECM) components inside the TME *via* many routes (2). Several pathways create a microenvironment that encourages tumorigenesis and metastasis by using soluble chemicals such as growth factors, cytokines, proteins, and metabolites (3, 4). Extracellular vesicles (EVs), such as small EVs (sEVs), have lately emerged as a potential mediator of information between both the tumour microenvironment (TME) and cancer metastasis (5, 6). Since the 1960s, multiple groups have demonstrated the functional nature of vesicles produced by diverse cells in culture. While platelet-secreted vesicles were revealed to regulate blood coagulation, it was discovered that EVs were capable of transporting trophic substances or nutrients to other cells (7–9). Moreover, several investigations found that secretory vesicles play a role in reticulocyte maturation *via* transferrin and receptor recycling (10, 11). Nonetheless, it was not until the late 1990s that multiple studies discovered how immune cell-derived EVs may act as antigen presenters and T cell stimulators by transporting MHC class I and MHC class II molecules on their membrane. During the first time, the study provided a separate channel for intercellular communication, emphasizing the importance of EVs in the immune system (9, 12, 13). TME components and EVs have a causal relationship, particularly in the context of EVs as a type of intercellular communication within the TME. Severe circumstances such as hypoxia, acidity, and food deficiency in the TME, which lead to cancer spread by changing EV release from cancer cells, are a clear illustration of it. Additionally, non-malignant cells in the TME produce EVs, which can influence cancer dissemination by impacting cancer protrusion, invasion, cell proliferation, altering ECM, and avoiding anti-tumor immune response. After understanding the biological causes of EVs mediated metastasis, researchers have now identified novel techniques for using EVs to avoid metastasis and fight cancer. The most exciting aspect in this respect is the use of EVs as an identifying guide for aberrant development. The greater quantities of disseminated EVs in cancer patients relative to normal people may be useful in identifying possible biomarkers (14, 15). Profiling EV cargo for certain miRNAs and proteins can further increase EV characteristic capability. Recently, the utilization of EVs for medicinal purposes has been investigated. Therefore, in this review we shall discuss or concentrate on the most recent findings in EV biology in connection to cancer development and the TME.

## Nomenclature and biogenesis of EVs

EVs are membranous vesicles released by a variety of cell types. Exosomes (small EVs), ectosomes, Microvesicles (large EVs) apoptotic bodies, big oncosomes, and migrasomes are all types of EVs. EVs are diverse in terms of their origin, manner of release, size, and biochemical composition (**Table 1**). Currently, EVs are categorized based on their mechanism of release or size. EVs can be released by “donor” cells by outward budding of the plasma membrane, which is known as shedding microvesicles (MVs) or ectosomes (16).

**Table 1:** Classification of EVs.

Type of EVs	Size (nm)/Shape	Biogenesis	Cargo content
Small EVs/Exosomes	30-100nm, Spheroid	They carrying ILVs are generated by ESCRT machinery and merge with the plasma membrane for release. Bascially early endosomes mature into late endosomes	HSPs, Tetraspanins (CD9, CD63, CD81), Biogenesis components (ALIX, TSG101) and integrins.
Large EVs/Microvesicles	100-1000, irregular	Membrane outward budding, followed by fission *via* contractile machinery	Integrins, selectins, CD40 ligand, fotillin-2, Death receptors (CD40) adenosine diphosphate ribosylation factor 6, phosphatidylserine, VCAMP3, ARF6
Exomeres	~50nm, spheroid	non-membranous nanoparticle isolated from the small EVs	High concentration of metabolic enzymes and hallmark related proteins in glycolysis and mTORC1 signalling
Apoptotic bodies	>1000, variable	During planned cell death, cytoplasmic fragmentation occurs.	Annexin V, Caspase 3, Phosphatidylserine and histones.
Oncosomes	>1000	Cell body cleavage to large cytoplasmic extensions	Cell adhesion molecules (CD44, integrins, ICAM), cytokeratine 18, CD9, CD81.

Another mechanism for sEVs release is the inward budding of the endosomal membrane, which results in the creation of multivesicular bodies (MVBs), with sEVs released by fusing of the outer MVB membrane to the plasma membrane (17, 18). Even though variables are used, lEVs have diameters ranging from 50 to 10,000 nm, while sEVs have diameters ranging from 30 to 150 nm (19–21). Overall, EVs are made up of a wide range of vesicles ranging in size from 30 to 1000 nm and carrying a variety of cargos, and the size distributions of the various types of vesicles overlap.

The diverse character of secreted nanoparticles is being more appreciated (22, 23). A form of tiny (50 nm) non-membranous nanoparticle known as an exomere was discovered recently *via* asymmetric flow field-flow fractionation (AF4). Exomeres have a high concentration of metabolic enzymes and hallmark related proteins in glycolysis and mTORC1 signalling (23). Exomeres selectively produce proteins, nucleic acids, and lipids in conjunction to proteins. Furthermore (24), Zhang et al., 2019 reported that exomeres are functional, since they contain both b-galactoside a2,6-sialyltransferase 1 (ST6GalI), which adds a2-6 sialic acid to N-glycosylated proteins, and the Epithelial Growth Factor Receptor (EGFR) ligand, amphiregulin (AREG). ST6Gal-I is transported from exomeres to recipient cells and sialylates cell-surface proteins such as b1-integrin. This is relevant in light of ST6Gal-pro-neoplastic Is activity and the importance of integrins in metastasis regulation (25–32).

Due to their potential to transport biomolecules between cells (33–36) and impact the extracellular milieu *via* control of essential nutrients, both kinds of EVs are considered as mediators of cell-to-cell communication (37, 38). EVs not only perform physiological duties including neurotrophic support (39, 40) and the removal of undesirable cellular components (41), but they also play pathophysiological roles in inflammatory and degenerative illnesses (42–44). As a result, as recently proposed by Tricarico and colleague, blocking the synthesis and release of EVs may be an important therapeutic target (45). In recent years, there has been a lot of interest in the process of EV biogenesis. We suggest readers to read these other good reviews for discussion of the involvement of proteins in exo- and ectosome biogenesis and fission, including members of the endosomal sorting complex needed for transport (ESCRT), small GTPases, and glutaminase (45, 46). Herein, we concentrate on the role of different type of EVs and their cargo content in the development of tumor and metastasis, which has gotten less attention.

## Factors influencing the tumor microenvironment and its progression

**Figure 1:**
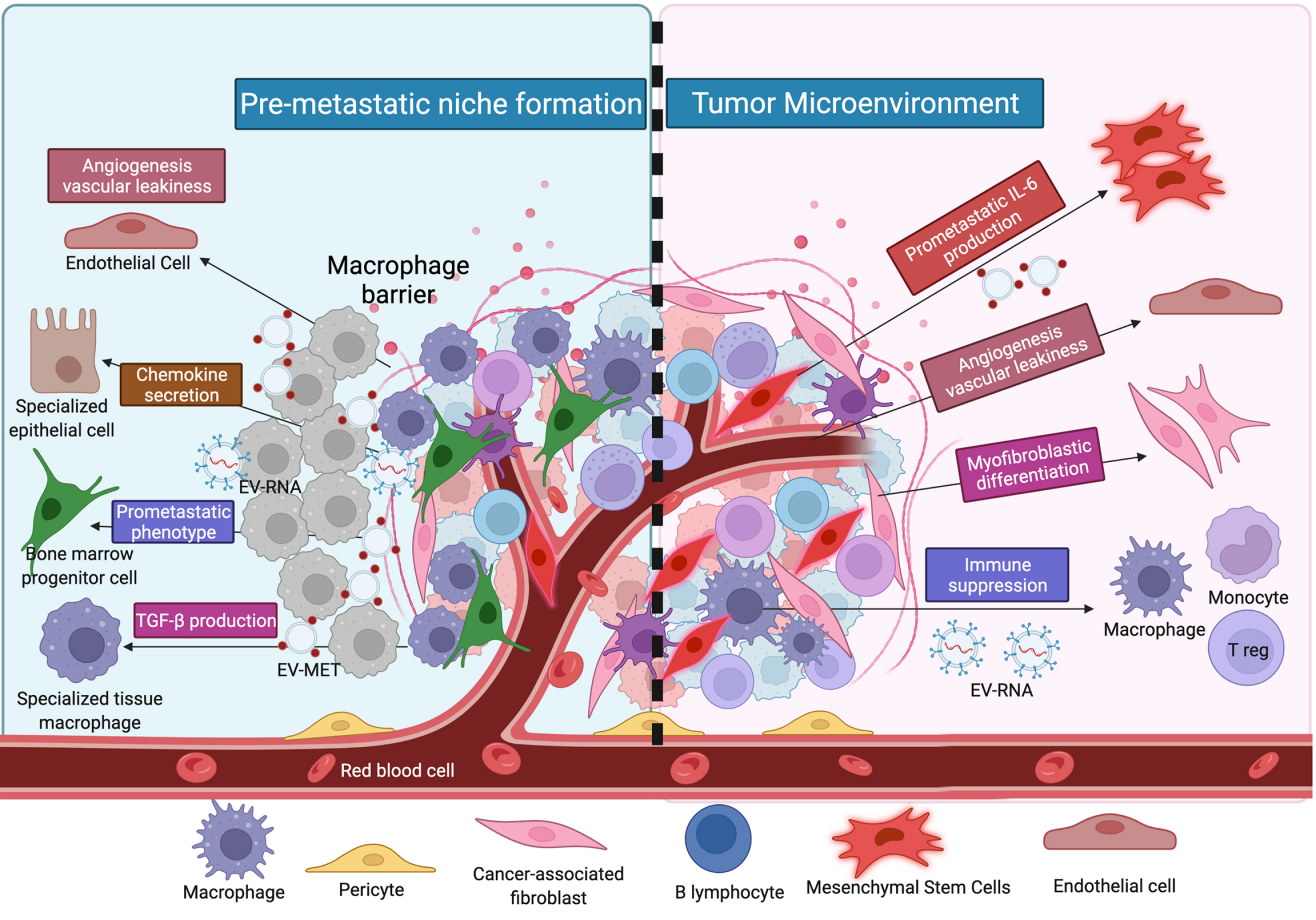
The illustration shows the distinction between the premetastatic and malignant niches. Hypothesis for cancer metastasis based on the notion of the metastasome; changing the milieu of target locations to make them hospitable hosts for alien DTCs, hence forming a premetastatic niche; and contributing to the proliferation of altered cells.

The primary changes in original cells that culminate in creation of a tumor is caused by mutations in oncogenes or tumor suppressor genes that originate in a normal cell and eventually lead to uncontrolled growth. The interplay of cancer cells with their “microenvironment” is analogous to the “seed and soil” relationship, referring to the TME’s strategic influence on disease genesis and progression because of its stimulatory or inhibitory signals (47–49). Immune cells, fibroblasts, fat cells, epithelial, endothelial, and mesenchymal stem cells, as well as soluble and insoluble molecules, extracellular matrix, and sEVs, are all major components of TME. Almost often, stromal cell glycolysis adaptation promotes tumor proliferation by the exchange of sEVs, which deliver metabolic intermediates such as pyruvate, lactate, glutamine, and ketones to cancer cells, which cancer cells may employ to create macromolecules. (**Figure 1**). In this context, fibroblasts, which make about one-third of stromal cells, play a critical role in cancer development. Despite the fact that several research have focused on the modulatory effect of soluble factors on the TME, fresh evidence has recently been revealed regarding the possible role of sEVs in regulating the TME and promoting aggressive tumor behaviors. Synergy between cancer cells and stromal cells such as bone marrow-derived cells (BMDCs), cancer-associated fibroblasts (CAFs), tumor endothelial cells (TECs), and tumor-associated macrophages (TAMs) is required for cancer cell survival and progression inside the TME (14, 50, 51). EVs formed by various tumor microenvironment cells have been shown to impair the host immune system in studies. These vesicles carry signals that affect the activation, apoptosis, proliferation, and metabolism of immune cells such as dendritic cells, T cells, monocytes, and natural killer cells, hence promoting tumor development and survival (12, 51).

Cells have been recognized for many years to be able to release large EVs (lEVs), which are regarded as one of the most essential physiological endogenous carriers for delivering molecular information among cells. The quantity of lEVs generated by cells appears to correspond with their “state of activation” and may be in reaction to local changes in the tissue milieu (52).

EVs are a wide population of naturally occurring membrane vesicles ranging in size from nano to microns that are released by practically all cell types. They have emerged as a unique and essential participant in intercellular communication, owing to their capacity to transport biological material to recipient cells, which includes proteins, lipids, and nucleic acids (11, 15, 53). It is becoming clear that EVs play a significant role in the control of physiological processes such tissue healing, stem cell maintenance, and coagulation. EVs have established themselves as essential participants in pathophysiological processes in illnesses such as cancer, neurological disease, and viral infection. Additionally, they are members of a large family of lipid bilayer-derived vesicles that are discharged by practically every live cell. sEVs are the EVs of intracellular communication. Additionally, sEVs serve as a “middle man” in the transport of biological macromolecules, triglycerides, and organic compounds within a biological body. The production of sEVs is a dynamic yet strictly regulated biological process. It is becoming increasingly clear that oncogene trafficking *via* sEVs can affect the function of cellular signaling pathways in targeted cells, hence playing a critical role in carcinogenesis, proliferation, and metastasis (54, 55). Whenever EVs or sEVs are gobbled up by targeted cells, they may partially or completely alter the cells’ state. The EV-cargo of pre-transformed stem cells transfer alters the recipient cell by affecting its functionality. It is still uncertain if EVs may cause long-term genomic changes such as mutation generation, although it has been revealed that several oncogenes can not only acquire incorporated into EV-cargo, but can also promote EV synthesis (56).

sEVs produced from melanoma stimulated endothelial cells dose-dependently and enhanced endothelial spheroid building, as one piece of evidence. They created a unique three-dimensional (3D) *in vitro* angiogenesis model to investigate the function of sEVs in tumor development (57, 58). Hood and colleagues revealed that sEVs can stimulate the creation of endothelial tubules and endothelial spheroids, changing the tumor microenvironment and boosting tumor growth. Melanoma-derived sEVs were discovered to pre-condition sentinel lymph nodes for tumor spread, since melanoma cells prefer to move to areas rich in melanoma-derived sEVs (59). The distribution of the oncogenic genotype *via* EVs generated by cancer cells effects heterotypic cells in the milieu, notably endothelial cells, fibroblasts, and lymphocytes, during cancer progression. For example, EVs produced from cancer cells overexpressing wild-type EGFR can activate the vasculature by delivering the antigen to adjacent endothelial cells, resulting in the production of Vascular Endothelial Growth Factor (VEGF) and subsequent autocrine signaling *via* its receptor Vascular Endothelial Growth Factor Receptor (VEGFR2) (60). Similarly, in colorectal cancer cells, tissue factor (TF) expression (a primary cellular initiator of blood clotting and a signaling component of angiogenesis and metastasis) relates specifically to the cells’ genetic state, such as an enabled KRAS gene or a p53 mutation that impairs angiogenesis and growth capacity *in vivo*. The investigators demonstrated for the first time *in vivo* that homing of sEVs from metastatic tumor alone is sufficient to establish a pre-metastatic milieu in sentinel lymph nodes, conducive sEVs to the site of tumor cells. Metastasis and significant death rates ensue from this anti-inflammatory phenotype. sEVs communication happens in both directions at the same time: immune cells release sEVs that reach cancer cells and vice versa (61–63).

EVs released by hypoxic squamous carcinoma cells have the ability to alter the microenvironment and promote angiogenesis and metastasis (64, 65). Additionally, there are three major hypotheses on how EVs enable intercellular communication: (i) Proteins contained within vesicle membranes have the potential to function as ligands for receptors on the surface of receiving cells. Additionally, proteases may degrade some of these membrane proteins, converting them to soluble versions. Membrane proteins in their soluble form may interact with cell surface receptors. (ii) EVs can be absorbed by recipient cells by fusing their membranes with the cellular membrane, therefore releasing their contents into the cytoplasm. (iii) Endocytosis (pinocytosis and/or phagocytosis) is a mechanism *via* which EVs enter the recipient cells (56, 66). Contact between the receptor and the ligand most likely initiates a signaling cascade, whereas internalization of EVs into recipient cells results in the transfer of the EVs payload, activating a range of downstream events (67, 68). Certain growth-regulating genes, like as Myc, Warts/Hippo, and p53, are engaged in cell competition, suggesting that they contribute to there homeostatic mechanism (69). EVs produced from transiently transfected tumor cells have been demonstrated to transport plasmid DNA (pDNA) encoding reporter molecules to recipient cells while expressing no messenger RNA. This result indicates a possible share mechanism of EVs mediated pDNA and gDNA transfer. Further investigation of the mechanism will advance our understanding of cell-cell communication via EV mediated DNA transfer. As a result, it is possible that the transfer of pDNA was facilitated by the same mechanism as the transfer of genomic DNA fragments, implying that understanding the underlying mechanism of EV-mediated transfer of pDNA and genomic DNA fragments will advance our understanding of cell-to-cell communication *via* DNA contained in EVs (70).

Among all non-coding RNAs (ncRNAs), long non-coding (lncRNAs) has recently been discovered in sEVs and linked to cancer (71, 72). Similarly, to microRNAs (miRNAs), the quantity of lncRNAs varies across cells/sEVs and between healthy and pathologic situations (71, 72). Tumor liquid biopsies have evolved as a non-invasive method for identifying possible biomarkers for cancer prediction, diagnosis, and progression (73). Numerous studies have established the presence of nuclease-resistant extracellular miRNAs in all recognizable physiological fluids (74). Since then, growing evidence indicates that extracellular miRNAs can be protected from RNAse degradation by either (1) encapsulating them in lEVs such as apoptotic bodies, shedding vesicles, and sEVs, or (2) complexing with AGO proteins. The bulk of miRNAs identified in bodily fluids lack lEVs and are coupled with Ago family proteins, which appear to be highly stable in protease-rich environments (74–76).

### Interplay between the tumor microenvironment associated cells

Recent research has uncovered another mechanism by which tumor cells elude identification by NK cells and hence hinder the NK-mediated immune response. Indeed, tumor cells synthesis vesicle-bound chemicals (cytokines, NK cell receptor ligand-NKG2D ligands, and miRNAs) that selectively target and inhibit NK cell function (77). Additionally, miRNAs delivered by carboxyl/cholinesterases (CCEs) may operate as ligands, attaching to Toll-like receptors and inducing inflammation. Indeed, it has been proven that the oncogenes miR-21 and miR-29a, which are generated from sEVs from lung cancer cells, interact with murine and human Toll-like receptor (TLR). sEVs, on the other hand, may contribute to the particular activation of T lymphocytes against cancer cells by transporting membrane proteins found on cancer cells, such as HER2/Neu (78–80).

Mesenchymal stem cells (MSCs)derived EVs include a diverse array of non-coding RNAs, including miRNAs. miRNAs are short non-coding RNAs that precisely target certain messenger RNAs in order to regulate post-transcriptional gene expression. It has been demonstrated that EVs produced from a range of cell types possess distinct miRNA patterns that may be transmitted to target cells (81, 82). EVs produced from murine-MSCs were also demonstrated to dramatically reduce VEGF synthesis in breast cancer cells, hence decreasing angiogenesis *in vitro* and *in vivo* (83–85). Tumor frequency and growth significantly enhanced when human gastric and colon cancer cell lines (SGC-7901 and SW480, respectively) were mixed with MSCs or MSC-derived EVs and subcutaneously fed to nude mice (86, 87). This impact was ascribed to an increase in proliferating cell nuclear antigen (PCNA) positive cells in tumor, which is a sign of enhanced cancer cell proliferation *in vivo*. There has been no indication that EV stimulated cancer cell proliferation *in vitro*, and there was no variation in the number of cells in the G0/G1, S, or G2/M phases compared EV-treated and untreated cells (88).

It has been proven that the cargo of the EVs produced from MSCs contain various proteins and miRs which fight cancer. Contradictory effects found in various tumor types may be due to the complexity of the systems involved. It is crucial to determine which substances transported by EVs have the potential to disrupt these pathways and, as a result, which cancer types may benefit from MSC-EV therapy. A group of researchers discovered that MSCs generated by cancer stem cell (CSC) EVs were capable of boosting *in vivo* tumor growth by enhancing proliferation and vascularization in one of the experimental conditions. On the other hand, unstimulated MSCs were unable to drive tumor formation, highlighting the importance of pre-conditioning MSCs in the tumour microenvironment (89, 90). Numerous studies have established that tumor-EVs communicate with surrounding cells, hence producing an environment favorable for tumor formation. Wysoczynski and Ratajczak discovered that EVs produced by lung cancer stimulate angiogenesis by altering stromal cells and enhancing the expression of a variety of pro-angiogenic proteins (as IL-8, VEGF, OSM, MMP9). Through the secretion of EVs, CSCs may play a significant role in the tumor micrenvironment niche defense (82, 85). The effects of CSC-EVs on MSCs demonstrate that these vesicles are engaged in the communication between tumor and stromal cells (89, 91).

The role of EVs in tumor interaction, particularly with MSCs, is crucial for anti-tumor treatment. MSCs’ antitumor activity varies according to the type or stage of developed tumor. While naïve MSCs may have antitumor activity, MSCs preconditioned with tumor EVs may exhibit phenotypic changes and promote tumor formation (81). One proposed technique for overcoming this situation is to pharmacologically limit the release of tumor EVs prior to the delivery of MSCs, hence avoiding their deleterious consequences (92).

In essence, the data from the previous paper indicate the importance of EVs in tumor-MSC interactions. CSCs, in particular, have the ability to affect the phenotypic of MSCs *via* EV secretion. MSCs that had been changed became more susceptible to tumor chemo-attractive stimuli, facilitating tumor cell migration and proliferation, and perhaps facilitating tumor vascularization (89, 93). Even after stimulation was stopped, the phenotypic changes persisted, indicating a long-term shift in MSC phenotype. sEVs have been implicated in the regulation of immunological responses, EMT, activation of cancer associated fibroblasts (CAFs), and angiogenesis in a number of investigations. CAFs also produce sEVs, which have the ability to change cellular metabolism. These sEVs have the ability to suppress mitochondrial oxidative phosphorylation, causing cancer cells to switch to glycolysis and glutamine dependent reductive carboxylation (82, 84). The interactions of cancer-derived EVs with fibroblasts have garnered considerable interest. EVs generated from chronic lymphocytic leukemia were actively integrated into stromal cells. This resulted in the secretion of inflammatory cytokines by cancer-associated fibroblasts (CAFs), creating a favorable environment for the tumor. EVs altered the phenotypic characteristics of fibroblasts in Hodgkin lymphoma and ovarian cancer cells, facilitating tumour development and progression. EVs impacted the proliferation and migration of pericytes in gastric cancer cells, indicating that they enhanced CAF marker expression in pericytes (94–96). miR-21, which was identified in EVs, was determined to be involved in the modification of normal hepatocyte stellate cells to CAFs. Additionally, clinical studies found elevated levels of miR-21, which is related with CAF activation and higher cell densities; hence, it may be involved in the development of hepatocellular carcinoma by encouraging tumor growth (97). miR-675 found in metastatic osteosarcoma-EVs is associated with reduced CALN1 expression in non-malignant fibroblast cells, enhancing their invasion and migratory abilities (98). In the context of EVs generated by pancreatic cancer cell lines, primary pancreatic fibroblasts from mice were transformed into CAF-like cells, a process regulated by miR-155 found in the EVs’ cargo (99, 100). miR-27a-expressing EVs have been shown to accelerate the transition of fibroblasts to CAFs in gastric cancer (101), In melanoma, miR-155-5p-expressing EVs have been shown to accelerate the transition of fibroblast cells to CAFs (94, 102), Similarly, miR-10b-expressing EVs from colorectal cancer cells have been shown to accelerate the transition of to CAFs (103), Human umbilical vein derived endothelial cells (HUVEC)-derived lEVs include functional peptide antioxidant components that allow them to function as Redox oxidative stress (ROS) scavengers. The antioxidant complement of MVs is not a multiple of the HUVEC machinery, but rather a separate complement engaged exclusively in the degradation of hydrogen peroxide and superoxide anion *via* the TRX-PRDX system (104, 105). HUVEC-derived lEVs include not only antioxidant enzymes and peptides, but also NADPH-synthesizing enzymes, indicating that they may have some level of self-protective antioxidant activity. Finally, when lEVs and HUVECs mature, their antioxidant machinery becomes more powerful, resulting in increased oxidative stress.miR-142-3p expressing EVs have been shown to accelerate the developed antioxidant system (e.g. HUVEC). The cargo of TEX (tumor-derived sEVs) comprises components that trigger immune cell malfunction in various ways, suppressing the antitumor immune response (106, 107). TEX first interacts with immune cells through ligands or antigens recognized by lymphocytes *via* cognate receptors. TEX bind to the surface membrane and are then absorbed into the cytoplasm *via* receptors. By contrast, phagocytic cells such as macrophages and dendritic cells rapidly absorb and ingest TEX (108). T cells do not appear to readily internalize TEX; rather, TEX appears to interact with surface molecules, transducing signals that result in prolonged Ca^2+^ influx and activation of downstream signaling molecules, so modifying the recipient cell transcriptome. Correlations between the molecular and genetic profiles of TEX and their immunosuppressive effects are being established, as are thorough investigations of the TEX transcriptome and proteome.

## Signaling content of EVs in metastatic niches of various organ specific tumor

### Gastric and pancreatic cancer

sEVs pathways may occasionally discard tumor-suppressive miRNAs that hinder metastatic development. For example, it was discovered that sEVs derived from metastatic gastric cancer cells expressed the let-7 miRNA family at a higher level than sEVs derived from non-metastatic parental cells, implying that metastatic cells may use the sEVs mechanism to eliminate tumor suppressor miRNAs, thereby strengthening their aggressive behavior and signaling by sEVs microRNAs in Cancer. Ohshima et al. also discovered that the let7 miRNA family is prevalent in sEVs isolated from a metastatic gastric cancer cell line, and that this miRNA family may be linked to the cell line’s oncogenic and metastatic potential (109, 110).

**Figure 2:**
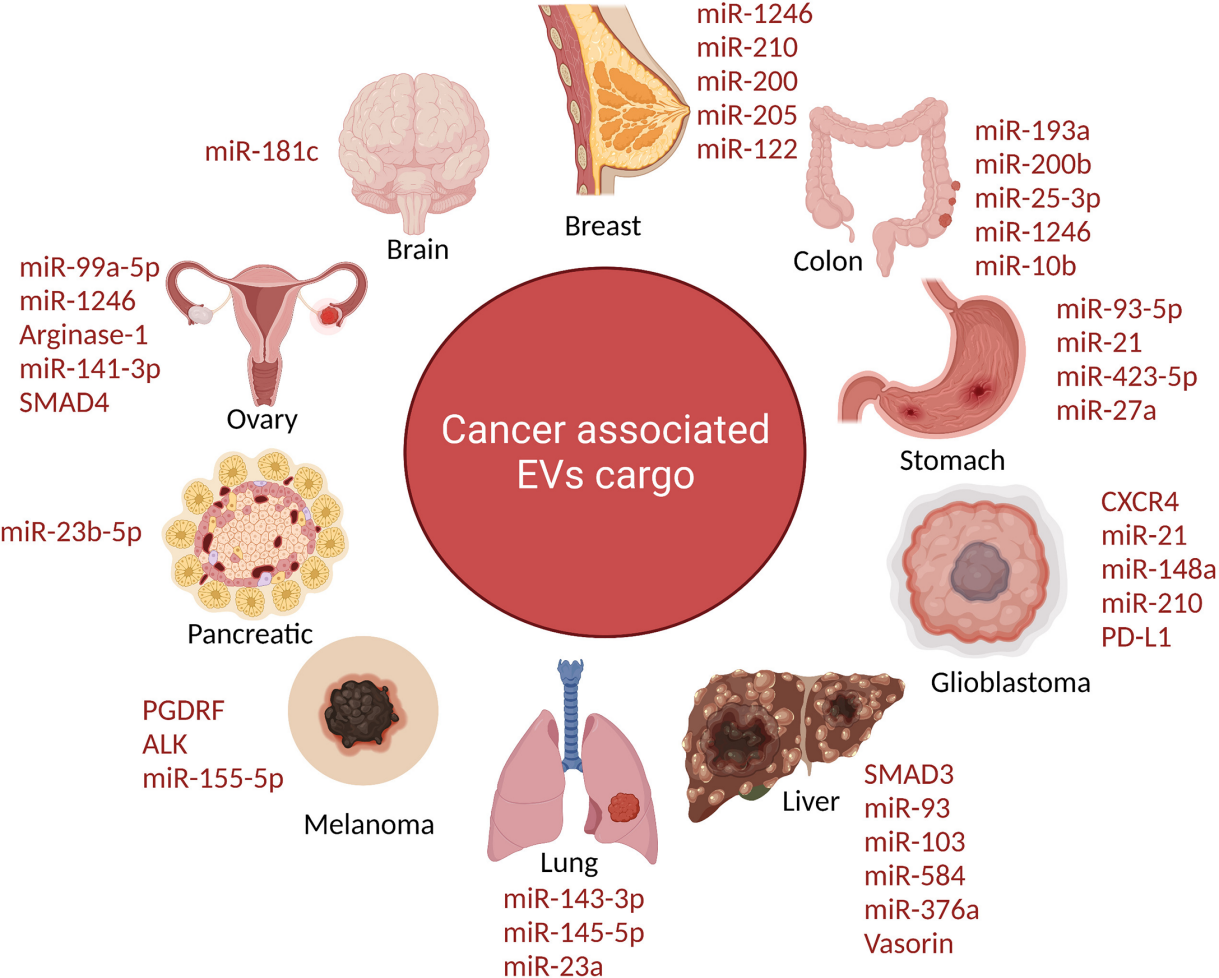
The schematic diagram depicts the extracellular vesicles derived from tumor contain distinct protein cargo which promote tumor progression. Tumor-derived EVs carry a specific protein cargo that aids tumour development. Components in the cargo of TEX (tumor-derived sEVs) cause immune cells to fail in diverse ways, inhibiting the antitumor immune response. TEX initially interacts with immune cells *via* ligands or antigens that lymphocytes identify through corresponding receptors. TEX binds to the surface membrane before being absorbed into the cytoplasm through receptors.

Another recent study revealed that metastatic bladder cancer cells have elevated amounts of miRNAs with tumor-suppressive capabilities (e.g., suppression of invasion, angiogenesis, and pulmonary metastasis), including miR-23b, miR-224, and miR-921 (111) (**Figure 2**).

### Breast & ovarian cancer

Breast cancer the amount of focal adhesion ikiki and EGFR in plasma fractions, as well as the number of lEVs, were associated to distinct stages of breast cancer. Let-7a was delivered intravenously to mice with EGFR-expressing breast cancer xenografts *via* miRNA-loaded sEVs co-expressing GE11 peptide on their surface. *In vivo* imaging demonstrated that sEVs expressing GE11 move preferentially to tumor tissue, while *in vitro* studies confirmed that silencing EGFR decreased targeted sEVs uptake. Although xenograft development was dramatically decreased in mice treated with GE11/let-7a sEVs, giving the first direct proof of therapeutic usefulness, let-7a controlled gene expression was not altered as compared to control sample (112, 113). Tspan8 has been linked to a variety of cancer types, including pancreatic, colorectal, esophageal, and melanoma. This is the first research to investigate Tspan8 expression and function in breast cancer (114, 115). The majority of initial breast cancer lesions and metastases to the brain, bone, lung, and liver contained Tspan8 protein. Tspan8+ tumors developed many liver and spleen metastases in a syngeneic rat breast cancer model, but Tspan8 tumors had a much-reduced predisposition for metastasis, showing that Tspan8 plays a role in metastases. They found that Tspan8 promotes E-cadherin upregulation and Twist, p120-catenin, and b-catenin target gene downregulation, resulting in a phenotypic shift akin to that observed during the mesenchymal–epithelial transition (116). Additionally, Tspan8 + cells exhibited increased cell–cell adhesion, decreased motility, and a reduced susceptibility to irradiation. Tspan8, a transcription factor that regulates the content and function of EVs, permitted a multifold increase in the quantity of EVs in cell culture and in the blood of tumor-bearing animals. E-cadherin and p120-catenin protein levels were elevated in these EVs, and Tspan8 and p120-catenin immunoprecipitated together, suggesting that they may interact (117). In summary, it was established that Tspan8’s existence in primary breast cancer lesions and metastases, as well as its role as a regulator of cell behavior and EV release in breast cancer. Ovarian cancer patients differ in their protein and miRNA composition from those from healthy individuals; the number of circulating sEVs in patients was four-fold that of healthy individuals (**Figure 2**).

sEVs from cultivated cell lines and malignant ascites include EpCAM and CD24 (118). Zhao and colleagues subsequently created ExoSearch, a microfluidic device that used anti-CA-125, anti-EpCAM (Epithelial Cell Adhesion Molecule), and anti-CD24 antibodies to collect sEVs from plasma of healthy controls and patients with ovarian cancer (cluster of differentiation 24 or heat stable antigen CD24) (119–121). Studies have proven that EVs produced after cisplatin treatment can have a variety of impacts on recipient cells. Adaptive responses include a rise in medication resistance and invasiveness. Interfering with EV transfer between cells may be a way for increasing tumor susceptibility to chemotherapy, but further research is needed to see whether these advantages can be applied to the *in vivo* context.

### Prostate cancer

Proteomic analyses of PCa EVs on several PCa cell lines and clinical tissues have been published. EVs from individuals with prostate cancer typically include cancer-related proteins such as CD9, CD81, and TSG101, as well as Annexin A2, Fatty Acid Synthase (FASN), and a prostate cancer-specific biomarker termed FOLH1 (Prostate Specific Membrane Antigen or PSMA) (122). PSMA is a transmembrane glycoprotein that is secreted by normal prostate epithelial cells and is increased in androgen-depleted prostate cancer. It has also been shown to be strongly induced in poor prognosis, metastatic, and hormone-refractory carcinomas. EVs produced by hypoxic PCa cells carry information that could be directly linked to latent PCa cell invasiveness and motility. EVs produced by diverse PCa cells also convey a number of functional proteins to recipient cells that do not have these proteins at the start (123). EVs derived from PC-3 (6 and 3 integrins) and CWR22 (3 integrin) cells have also been demonstrated to shuttle integrins to DU-145 and C4-2B cells that do not express integrins ordinarily, hence promoting progression and invasion. The androgen-dependent TMPRSS2-ERG gene fusion can be identified in 50% of clinically localized PCa and 90% of PCa overexpressing ERG (124). EVs obtained from VCaP contain the TMPRSS2-ERG RNA (an androgen responsive cell). In urine-derived EVs (CD63-labeled vesicles), both TMPRSS2-ERG and a prostate cancer biomarker, Prostate Cancer Antigen (PCA-3) mRNA, were found, indicating that the mRNA composition of EVs is informative and may give prospective prostate cancer biomarkers (**Figure 2**) (125).

### Lung cancer

PD-L1 is identified on the surface of EVs isolated from NSCLC patients’ plasma, and the number of PD-L1-positive EVs is proportional to the degree of PD-L1 expression in the same patient’s tumor tissue (126). PD-L1-expressing EVs generated from NSCLC cells can cause T lymphocyte death, increasing tumor development in mice. In this context, it looks as though these EVs prevent Jurkat T cells from producing INF-γ, so establishing an immunological inactivation circuit (127). sEVs express miR-205-5p, miR-483-5p, miR-375, miR-200c-3p, miR-429, miR-200b-3p, miR-200a-3p, miR-203a-3p, and miR-141-3p that were retrieved from the pleural fluid of lung cancer patients (128–131). EVs provide insight into cell physiology and disease. These EVs, derived from pleural fluid, are linked to lung adenocarcinoma and have high plasma levels, which could be useful in detecting the presence of tumors and their response to therapy. The activation of the microenvironment is intimately linked to EMT. sEVs produced from advanced lung cancer can trigger vimentin expression and EMT in human bronchial epithelial cells (HBECs), and these TDEs can promote cell migration, proliferation, invasion, and metastasis. In normal cells, ZEB1, a master EMT transcription factor, may produce a mesenchymal phenotype … In NSCLC patients, circulating interferon- (INF-γ) in the tumor microenvironment promotes the production of immunosuppressive EVs (132, 133). Tumor-derived EVs have distinct integrin patterns that lead them to favored organs, where they can influence the premetastatic environment’s organotropism. Similarly, when ALK-mutated patients are treated with currently available medications like as crizotinib, ceritinib, and alectinib, a sequence of on-target and off-target resistance mutations can arise, which ctDNA screening generally misses. Through the transmission of oncogenic miRNAs, EVs can serve as a vector for the emergence of treatment resistance. sEVs enriched in certain miRNAs released by gefitinib-resistant cell lines, for example, can impart resistance phenotypes to recipient cells (134). The lung is a common site of metastasis for many metastatic primary cancers, but the particular molecular process behind this tissue-specific metastasis is unknown. It is demonstrated that the lung microenvironment promotes the production of PMN, and that TEXs may play important roles in this process. Further research will clarify the mechanism of particular sEVs’ influence on tumor microenvironment and promotion of lung invasion, as well as that of other organs (**Figure 2**) (135).

### Cancer associated major EVs cargo

Glycolipid-based EVs were thought to play a role in normal cell signaling four decades ago. However, until recently, the exact nature, function, and biosynthesis of EVs were unknown. When multivesicular endosomes merge with the plasma membrane, they release EVs into the extracellular environment, as shown in reticulocyte formation (71). The group hypothesize that Tumor derived large EVs with malignant characteristics, known as metastasomes, induce a sequence of cell biological activities in different regions throughout cancer patients’ bodies, eventually leading to the formation of secondary lesions in susceptible areas (79, 87). Following the definition of the metastasome, they hypothesis two mechanistic models for tumour metastasis: the first, “tumor-organ-training (TOTr),” which entails altering the microenvironment of target sites to make them hospitable hosts for alien disseminated tumor cells (DTCs), thereby establishing a premetastatic niche; and the second, “tumor-organ-targeting (TOTa),” which entails contributing to the propagation of the transformed (86, 94, 99). Moreover, there’s evidence that EVs generated by tumors include DNA fragments that might make up the entire genome.

**Figure 3:**
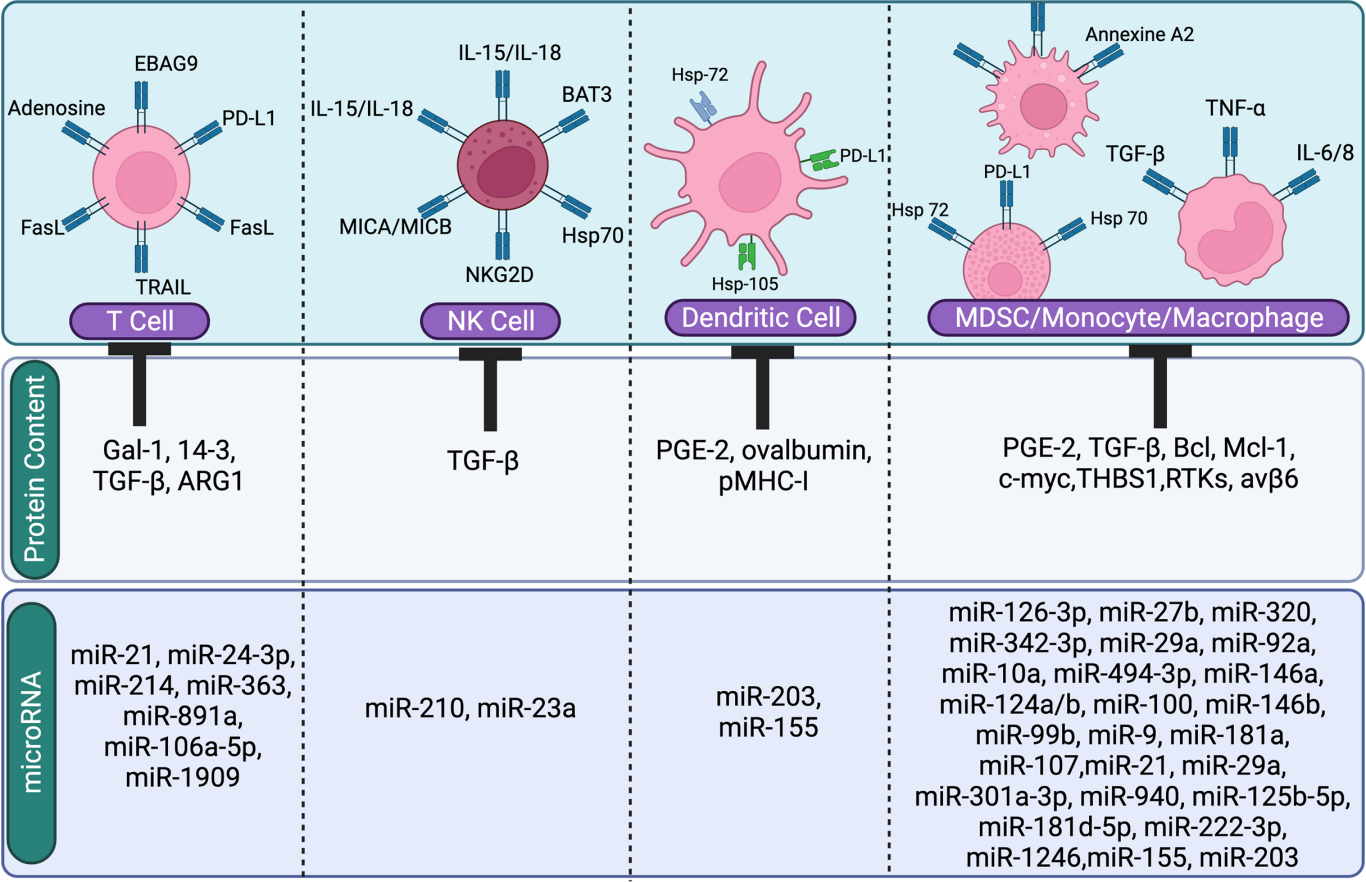
The image shows the suppressive role of tumor derived EVs content (miRNA or protien) on immune cells. Tumor-derived EVs regulate and inhibit immune cells (T cells, NK cells, Dendritic cells, and macrophages).

The presence of PTPRZ1 (Protein Tyrosine Phosphatase Receptor Type Z1) –Met (PTPRZ1-Met) in EV cargo in glioblastoma cells led in an aggressive phenotype when transferred to corresponding cells (136). While EVs were delivered to susceptible cells, anaplastic lymphoma kinase-associated mRNAs activated the MAPK signaling pathway. Acquiring glioma cells lacked this isoform, thus they accumulated EVs expressing EG-FRvIII (a truncated carcinogenic version of the epidermal growth factor receptor) and activated the MAPK and AKT pathways (29, 59). WNT signaling was enhanced in recipient cells by the oncogenic mutant-catenin (EV cargo), leading to colorectal cancer development. Furthermore, EVs in ovarian cancer showed EV-mediated SMAD 4 mutations. TGF-β Derived sEVs (TDEs) in the lungs can be taken up by macrophages, allowing tumour growth and immunological suppression. By interacting with Fas/FasL, TDEs can release transforming growth factor beta (TGF-β) to promote regulatory T cell proliferation and trigger effector cell death (137). TGF and interleukin (IL) may also control tumour cell migration in the lung. sEVs allowed drug induced COX-2 overexpression to be transmitted from lung cancer cells to neighboring cells, resulting in TDE induced elevation of PGE-2 and VEGF in TDE binding cells and production of an inflammatory response (138) (**Figure 3**).

#### Different types of EVs and their roles in the light of biomarkers identification

Although potential biomarkers for early cancer diagnosis can be found in a variety of biological fluids, blood is the biological fluid that contains the most biomarkers and is also the most easily accessible. Serum proteins, free nucleic acids, and metabolites are some examples of potential candidates for such biomarkers (139–141). Several studies found serum and plasma proteins, the quantities of which are connected with an increased risk of several types of cancer. In addition to circulating tumour cells (CTC) and circulating free DNA (cfDNA) are another potential biomarker for lung cancer (142, 143). In the more recent past, serum metabolites and lipids have emerged as another potential class of biomarkers in the detection of cancer. However, despite the fact that a large number of potential biomarker indicators have been proposed up until this point, only a small number of those indicators have been successfully verified in the appropriate clinical settings. The lack of sensitivity and analytical repeatability was the primary cause, which ultimately resulted in the rejection of possible candidates from further phases of biomarker testing. Due to the fact that EVs are an invasive source of biomarker, they turned out to be a blessing in disguise in this scenario. Biomarkers are used to diagnose many types of cancer. There are a few publications out there that confirm the utilization of Evs as a biomarker in the identification of cancer. sEVs include neurodegenerative disease linked proteins including the prion protein, β-amyloid and α-synuclein. Primarily established *in vivo* with prion disease, exosomes are postulated to also facilitate the migration of β-amyloid and α-synuclein from their cells of origin to the external environment (144). The organ of Corti is most likely responsible for the production of EVs that can be found in the inner ear. When ototoxic stress was applied to the EVs, a statistically significant decrease in the exosome protein levels as well as the number of particles per cubic centimeter was found. This may be interpreted as a lower cell quantity due to the extensive damage sustained by the hair cells, which is particularly prevalent in the cisplatin group. When the group that was given ototoxic medications was compared to the group that served as the control, it was found that the patterns of protein expression were different in both groups. The discovery that the significant hits in the proteomics analyses of the sEVs had previously been described in the context of hearing loss is an intriguing one. As a result, not only are sEVs changing in number and protein compositions, but they also appear to reflect the status of the inner ear hair cells. This makes EVs good candidates for use as biomarkers; however, additional research is required to fully describe the sEVs found in the inner ear and to establish their precise function there, particularly in the context of the use of ototoxic drugs (145–147). The several forms of EVs that circulate in body fluids are becoming an increasingly important source of cancer biomarkers. As a result, several different molecular components of serum- and plasma-derived EVs were investigated and shown to have varying concentrations in people with lung cancer and healthy individuals. Several research concentrated their attention on the miRNA found in these vesicles. Even though none of the proposed signatures of sEVs miRNA have yet to be clinically confirmed, the diagnostic potential of sEVs miRNA signatures is quite promising. The total number of miRNA species included in these signatures was somewhere around a dozen, and a few of those species were utilized in several signatures. It is important to highlight the fact that each of these miRNA species has a role connected to cancer and has been linked to the progression of lung cancer. In addition, a handful of them, including recognized oncomirs miR-17, miR-19, miR-21, and miR-221, emerged in many miRNA signatures of lung cancer based on both the total serum/plasma and serum/plasma-derived EVs (148). Another group demonstrated that macrophage migration inhibitory factor, or MIF, was significantly expressed in EVs formed from pancreatic ductal adenocarcinomas (PDAC), and that blocking MIF inhibited the establishment of a pre-metastatic niche in the liver as well as metastasis.

When they compared their findings with those of individuals whose pancreatic tumours did not grow, they discovered that the MIF was significantly higher in EVs taken from stage I PDAC patients who went on to develop liver metastases. According to their findings, EVs MIF may be a predictive sign for the development of PDAC liver metastasis and may also prepare the liver for the formation of metastases (149).

## Involvement of EVs in cancer metastasis

Communication occurs between the tumor and the stroma inside the tumor microenvironment. Currently, biogenesis and packaging of modified cancer EVs is not clear. It is still a topic of debate, whether the cells are packed abruptly or is this a regulated strategy for intracellular communication. On the other hand, cancer EVs have a profound effect on the behaviour of local or recruited stromal cells, forming a tumor-promoting microenvironment that promotes tumor angiogenesis, immunosuppression, and cancer cell acquisition of malignant features. Cancer EVs can trigger unique cellular responses by replicating the state of the original cancer cells, in addition to transmitting oncoproteins. Cancer EVs, for example, contain a large number of hypoxia-regulated RNA and proteins that boost endothelial cell activity and permeability (150–152).

Even though cancer EVs are tumor antigen carriers that have the potential to promote antitumor immunity, substantial evidence indicates that they mostly suppress the immune response. Tumor-derived EVs have been shown in multiple studies to decrease CD8+ cell proliferation and activation while increasing regulatory T cell development through unknown mechanisms. Cancer EVs can “train” innate immune components toward a pro-tumorigenic phenotype, in addition to their impact on the adaptive immune system. Furthermore, EV-associated microRNAs have been shown to polarise tumor-associated macrophages toward a pro-tumorigenic M2 phenotype.

### Role of stem cells derived EVs in cancer progression

Mesenchymal cells can also facilitate the pro-tumorigenic actions of cancer EVs. Webber and colleagues discovered that prostate cancer cells create EVs that cause myo-fibroblastic cell differentiation, resulting in increased angiogenesis and faster tumor development *in vivo* (84, 153). These effects were mediated by a membrane-associated form of TGF on the vesicle’s surface, and they couldn’t be replicated by giving stromal cells the soluble TGF counterpart. This study supports their previous findings that EVs generated by malignant bone tumor cells contain significant levels of membrane-associated TGF, which causes mesenchymal stem cells to produce IL-6. Injection of EV-educated MSCs into mice with bone tumors activated STAT3 and boosted metastatic spread, demonstrating that the inflammatory loop begun by cancer EVs inside the tumour microenvironment promotes tumor cell metastatic behaviour. EVs contribute to cancer development by acting on the extracellular matrix (ECM), in addition to their effects on local or recruited tumor-associated stromal cells (85, 154). According to Sung et al., tumor sEVs secrete an sEVs-bound version of fibronectin, which aids directed cell movement through tissue. The creation of focal adhesions is aided by the release of fibronectin-rich sEVs at the leading edge of migrating cells, which work automatically to stabilise leading edge indentations and speed up migration (155, 156). Tumor sEVs not only increase cell migration but also encourage directed movement toward a chemotactic gradient, according to the same research, but the exact processes underlying this behaviour are unknown.

Notably, tumor sEVs promote the formation and activity of invadopodium, promoting invasive cell behaviour. EVs secretion stimulates matrix disintegration by transporting the proteinase MT1-MMP to the plasma membrane, which increases the number and stability of invadopodia. Tumor-derived EVs have a tumor-promoting function that is not limited to the tumor microenvironment. Cancer EVs, on the other hand, enter the bloodstream and travel to distant organs, where they may encourage the growth of disseminated tumor cells. This process, known as pre-metastatic niche (PMN) generation, involves a number of discrete phases, including vascular leakiness, stromal component changes, and immune suppression (157). Costa-Silva et al. elaborated on this concept by revealing that pancreatic cancer EVs activate an intercellular signalling cascade that facilitates the development of pre-metastatic niches in the liver. High amounts of macrophage inhibitory factor (MIF) boost TGF synthesis in Kupffer cells, leading in hepatic stellate cell activation and subsequent fibronectin formation, according to the researchers. The fibrotic liver environment that develops attracts bone marrow-derived macrophages, speeding up metastatic development (149).

Additionally, the same group demonstrated that pancreatic cancer EVs activate an intercellular signalling cascade that promotes the establishment of a pre-metastatic niche in the liver. High amounts of macrophage inhibitory factor (MIF) in PDAC-EVs increase TGF synthesis in Kupffer cells, activating hepatic stellate cells and resulting in the formation of fibronectin, according to the researchers. The fibrotic liver environment that results attract macrophages derived from bone marrow, which promotes metastatic growth. To determine the proportional contributions of EV-mediated signalling and cancer cell intrinsic components to malignant cell organotropic activity, more research is needed. Cancer EV-associated proteins, as well as EV-enclosed small RNAs, all contribute to the formation of the pre-metastatic niche in different ways. miR-105, which is produced by breast cancer cells and targets the tight junction protein ZO-1, enhances vascular leakiness in distant organs, whereas miR-122 reduces glucose absorption by PMN stromal cells, boosting glucose availability and metastatic development (50, 100, 103). While these findings suggest that cancer EVs play an important role in the events leading up to cancer cells’ entry into future metastatic sites, their physiological significance must be confirmed. Indeed, most of these experiments have included exposing stromal cells to varying doses of cancer EVs or injecting tumor vesicles directly into the bloodstream. Considering physiological conditions, such as phagocytic cell internalisation, the amount of cancer EVs entering the circulation and accessing targeted (pre-metastatic) organs may be minimised. According to the researchers, melanoma EVs are captured and stopped from dispersing by a barricade of subcapsular sinus macrophages in tumor-draining lymph nodes. The authors shows that cancer progression and some anticancer drugs can break the macrophage barrier, allowing cancer EVs to enter the lymph node cortex and activate tumor-promoting humoral immunity. Earlier studies on EV-mediated interaction in cancer mostly focused on a unidirectional process in which tumor EVs influence the activity of stromal cells. Luga and colleagues discovered in 2012 that cancer cells may alter and recycle fibroblast-derived vesicles in order to activate an autocrine WNT-planar cell polarity signalling pathway, hence enhancing tumor cell motility and metastasis. Notably, the pro-tumorigenic activity of stromal EVs is usually determined by the generating cells’ exposure to cancer-derived signals (158).

For instance, tumor-associated macrophage polarization toward the M2 phenotype has an effect on the production of EV-associated oncogenic miRNAs that govern the invasiveness of breast cancer cells. Intriguingly, it was discovered that EVs produced from mesenchymal stem cells may have opposing response on multiple myeloma (MM)-progression. The tumor microenvironment may entirely weaken stromal EV function, since EVs derived from normal bone marrow-derived MSCs suppress cancer cell proliferation whereas EVs derived from multiple myeloma (MM)-MSCs promote tumor growth (159). The non-tumorigenic characteristic of naive MSC-EVs makes them an appealing cancer therapeutic option. As a matter of fact, MSC-EVs are a non-immunogenic, natural delivery pathway that has previously been employed to treat graft vs host illness in the clinic. Additionally, because these vesicles retain the MSCs’ surface expression profile, they may exhibit a similar affinity for tumor locales as their parent cells (160). Finally, a novel mode of EV communication involving mitochondrial DNA transfer has been associated with the development of treatment resistance. Sansone and colleagues discovered that EVs containing the entire mitochondrial genome generated by cancer-associated fibroblasts correct deficiencies in oxidative phosphorylation in hormone treatment-induced dormant breast cancer stem cells, resulting in dormancy release and resistance to hormone therapy (161). This fascinating work raises numerous important questions, including the identity of the EV subtype responsible for mitochondrial genome transfer. What happens when mitochondrial DNA is integrated into EVs? Most critically, how can mitochondrial DNA be transferred to the correct subcellular place and recipient cells restored to normal metabolic activity? Even though some parts of the discovery remain unknown, it reveals a potential metabolic communication pathway in cancer.

## A future prospects of EVs for biomarker identification and possible treatment in the field of cancer

Numerous researchers have investigated the natural role of sEVs as transporters of metabolites between donor and recipient cells and in initiating a biological response from a therapeutic perspective. sEVs have the potential to be employed as both tumor resistance mediators and medication delivery vehicles. Various studies depict the medicines proposed for tumor therapy depending on sEVs features. The development of a novel type of drugs that specifically target miRNA pathways is presently underway. They act yet by replacing synthetic or viral vector-encoded miRNA mimics for tumor-repressed miRNAs or by blocking oncogenic miRNAs *via* antisense mediated inhibition (50, 100, 103). Off-target effects, on the other hand, must be assessed before such medications can be safely provided in the clinic. Surprisingly, several preclinical investigations have shown that EVs may be modified to improve their capacity to target tumor tissues. Cellular EV absorption is believed to be cell type specific, although the processes involved in the process are not well understood. Furthermore, exogenously delivered EVs loaded with miRNAs should be subjected to dose escalation experiments in order to establish the therapeutic window and the maximum dosage permissible without saturating the endogenous miRNA processing machinery in non-tumoros cells.

**Figure 4:**
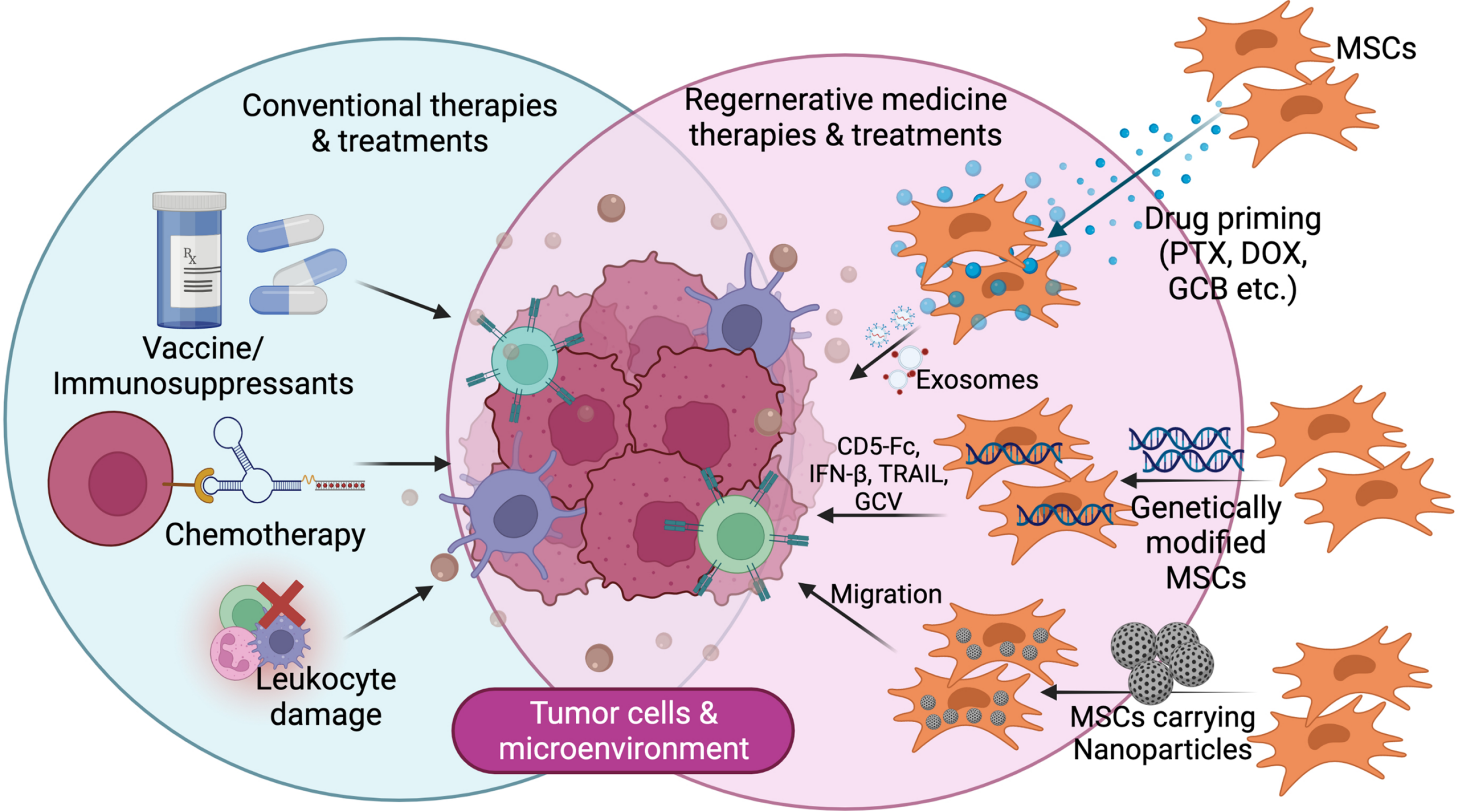
Futuristic line of cancer treatment focusing on tumor microenvironment with/using modified mesenchymal stem cells.

sEVs ability to heal neuronal disorders and brain inflammation in mice has paved the way for the creation of new cancer therapies (162). As medication carriers, sEVs have several advantages, including superior biodistribution and biocompatibility, as well as the capacity to cross biological barriers that are generally impenetrable, such as the blood-brain barrier. Furthermore, current advancements in medical nanotechnology and sEVs biology may allow for the loading of these vesicles with specific cancer medication combinations and the successful targeting of sEVs to the proper cancer cells (**Figure 4**).

Traditional transfection procedures were employed to successfully load genetic components into stem cell produced sEVs, such as anti-tumor mRNAs or siRNAs. sEVs produced by miR-146b-expressing stromal cells in bone marrow, for example, were collected by Katakowski et al. (163). In a rat model of primary brain tumor, direct infusion of these sEVs resulted in a substantial reduction in glioma xenograft growth. sEVs released by miR-122-expressing MSCs greatly improved the anticancer efficacy of sorafenib in a hepatocellular carcinoma tumor model in another investigation (164). Similarly, sEVs generated from MSC efficiently delivered siRNA to bladder cancer cells, successfully silencing the polo-like kinase 1 gene. Meanwhile, two techniques might be used to encapsulate small molecule medications inside sEVs. First, it was revealed that stem cells may swallow exogenous materials after being primed with them, bundle them into sEVs, and then release them into culture media *via* exocytosis. According to Pascicci et al., sEVs obtained from paclitaxel primed MSCs effectively reduced the development of a human pancreatic cancer cell line. Furthermore, these sEVs were reported to inhibit tumor development in leukaemia and myeloma cell lines. To prime MSCs, other medicines such as doxorubicin, gemcitabine, and cisplatin have been employed (165, 166). In reality, the drug content of sEVs is heavily influenced by the priming concentration, incubation period, and cell absorption method. Therapeutic medicines might instead be loaded into sEVs *via* the post-loading approach. sEVs were pushed to encapsulate medicines after being extracted from stem cell culture media through extrusion, electroporation, dialysis, or saponin-assisted methods. This enables the loading of both hydrophilic and hydrophobic drugs, as well as improved drug loading control and encapsulation efficiency. Recently, it was discovered that the lncRNA RP11-838N2.4 is up-regulated in erlotinib-resistant NSCLC cells. FOXO1 may be controlling this lncRNA’s expression by recruiting histone deacetylases to its promoter region. Furthermore, RP11-838N2.4 was substantially abundant in sEVs from erlotinib-resistant NSCLC patients (167). Removal of low molecular weight proteins (approximately 150 kDa) resulted in tumor shrinkage of 50% or more in six of the sixteen patient samples. Though lEVs were not identified, Marleau et al. believe that whole blood ultra-pheresis removed not only sEVs (which would have been unknown at the time), but also other EVs due to the high heterogeneity of EVs in circulation, and that this removal may have contributed to the observed tumor shrinkage (168). Aethlon Medical has developed a hemofiltration device that utilizes cartridges with a porous hollow fiber route and an affinity matrix that are compatible with continuous renal replacement therapy machines (169). Moreover, the contents of EVs mirror the contents of the cells from which they come (both stromal and tumor). EVs have the potential to be employed as biomarkers for diagnosis, as well as to predict or monitor a patient’s response to therapy. It has been established, at least in the instance of ovarian cancer, that miRNA profiling from circulating sEVs is equivalent to sEVs profiling from tissue (170–172). Tumor-derived sEVs in the peripheral circulation exhibit different miRNA expression patterns than healthy person sEVs. The idea of detecting early cancer alterations with a simple blood test is particularly appealing because venesection is a common and safe therapeutic procedure. However, while the detection accuracy of any screening strategy is critical for clinical translation, the fact that sEVs miRNAs are maintained within a lipid bilayer whilst actively circulating miRNAs are not implies that sEVs may be the greatest source for biomarker discovery. This demonstrates that sEVs’ miRNA composition is dynamic and may not exhibit consistent expression patterns over time. Nonetheless, Palma et al. showed that malignant cells produce sEVs miRNAs preferentially, and that the nature of sEVs alters in the malignant environment. As a conclusion, a relationship between sEVs content and EVs subtype may exist, which might be exploited in the clinic to give disease-specific diagnostic or prognostic indications.

## Conclusion

EVs play a role in cancer formation by transporting several forms of sEVs cargo to their target cells, and these bioactive chemicals activate a variety of oncogenic pathways in normal, malignant, and stromal cells inside the TME. By carrying specialized enzymes such as metalloproteinases, EVs might alter the microenvironment of tumor cells in favor of metastatic dispersal or implantation into specific organs. Blocking the dissemination of EVs using chemicals that link the vesicles to the vesiculating cells might potentially reduce tumor development or metastasis spread. On the other hand, screening for cancer genetic markers carried by EVs may enhance diagnostic procedures for specific malignant disorders. The viability of sEVs as prospective therapeutic agents is increased by understanding the key processes underpinning cancer aggressiveness *via* sEVs cargo trafficking allying PCa and stem cells in the TME. Although the chemicals involved in EVs formation have been examined, the extracellular and intracellular signals that regulate this procedure have yet to be discovered. Furthermore, the chemical processes were loaded with lncRNAs into sEVs that remains unknown. Furthermore, it is critical to investigate the mechanism by which each medication triggers sEVs production as well as the particular lncRNA sorting into subtypes of EVs.

## Author contributions

All of the authors named have made a significant, direct, and intellectual contribution to the work and have given their permission for it to be published.

## Acknowledgments

The authors would like to express their gratitude to the thousands of reviewers and experts who contributed to this unprecedented scientific and clinical crisis. We would also like to express our credit to BIORENDER, for the figures.

## Conflict of interest

The authors declare that the research was conducted in the absence of any commercial or financial relationships that could be construed as a potential conflict of interest.

## Publisher’s note

All claims expressed in this article are solely those of the authors and do not necessarily represent those of their affiliated organizations, or those of the publisher, the editors and the reviewers. Any product that may be evaluated in this article, or claim that may be made by its manufacturer, is not guaranteed or endorsed by the publisher.
